# Pregnancy and Neonatal Outcomes in Women Treated for Bowel Endometriosis: A Seven-Year Single-Centre Retrospective Matched Cohort Study

**DOI:** 10.3390/jcm13195956

**Published:** 2024-10-07

**Authors:** Vesna Šalamun, Gaetano Riemma, Tina Sirc, Eda Vrtacnik Bokal, Helena Ban Frangež

**Affiliations:** 1 Department of Human Reproduction, Division of Gynaecology and Obstetrics, University Medical Centre Ljubljana, 1000 Ljubljana, Slovenia; vesna.salamun@icloud.com (V.Š.); eda.bokal@kclj.si (E.V.B.);; 2 Obstetrics and Gynecology Unit, Department of Woman, Child and General and Specialized Surgery, University of Campania “Luigi Vanvitelli”, 80128 Naples, Italy; gaetano.riemma@unicampania.it

**Keywords:** endometriosis, colorectal endometriosis, surgery, pregnancy outcomes, in-vitro fertilization, bowel endometriosis

## Abstract

**Background/Objectives:** Deep infiltrating endometriosis has been linked to worsened maternal and neonatal outcomes. However, reports regarding bowel endometriosis are still scanty. We aimed to evaluate pregnancy, delivery, and newborn adverse outcomes in women after laparoscopic-assisted surgery for bowel endometriosis. **Methods**: A single-center retrospective cohort study was conducted at a tertiary-care university hospital. From January 2015 to December 2021, pregnant women who were diagnosed and treated for bowel endometriosis were matched using a 1:3 ratio with pregnant women with no history of endometriosis. Patients were matched using the Cox proportional hazards model to determine parity, age, BMI and gestational age-adjusted relative risk (aRR) with a 95% confidence interval (CI). Co-primary outcomes were the incidence of labor abnormalities and cesarean section (CS) rate. Co-secondary outcomes were incidence of complications related to pregnancy, delivery, and newborn. **Results**: A total of 71 pregnancies among women treated for bowel endometriosis and 213 from healthy controls were included. Patients requiring IVF/ET for getting pregnant were in the bowel endometriosis group relative to controls (43.7% vs. 11.7%; *p* < 0.001). Increased risk of labor abnormalities was present for bowel endometriosis relative to controls (21.1% vs. 17.4%; *p* = 0.040; aRR 1.39 [95% CI 1.06–2.05]). Risk of non-cephalic fetal presentation (14.1% vs. 6.1%; *p* = 0.016; aRR 3.08 [95% CI 2.03–4.68]), CS rate (43.7% vs. 24.9%; *p* = 0.003; aRR 1.75 [95% CI 1.23–2.49]), and emergent CS rate (19.7% vs. 8.5%; *p* = 0.009; aRR 2.21 [95% CI 1.55–3.16]) were significantly higher in women treated for colorectal endometriosis compared with controls. Moreover, placenta previa (9.9% vs. 0.0%; *p* < 0.001; aRR 21.82 [95% CI 2.19–116.40]), second-trimester hemorrhage (5.6% vs. 0.9%; *p* = 0.017; aRR 6.00 [95% CI 1.12–32.06]), postpartum hemorrhage (15.5% vs. 3.3%; *p* < 0.001; aRR 4.71 [95% CI 1.90–11.70]), and the need for transfusion during labor (5.6% vs. 0.5%; *p* = 0.004; aRR 12.00 [95% CI 1.36–105.60]) were increased in treatments vs. controls. Concerning neonatal outcomes, an increased risk for neonatal intensive care unit admission was seen in postsurgical endometriotic women relative to healthy controls (26.0% vs. 6.9%; *p* < 0.001; aRR 3.75 [2.04–3.86]). **Conclusions**: Women treated for bowel endometriosis seem more exposed to adverse pregnancy and neonatal outcomes relative to healthy controls. However, additional prospective and comparative studies are needed to validate the available evidence.

## 1. Introduction

Up to 50% of women who struggle with reproductive issues have endometriosis, which is a primary cause of both pelvic discomfort and infertility [1]. Deep infiltrating endometriosis (DIE) is the most severe form of endometriosis. Its traditional definition, describing a lesion with an infiltration depth of ≥5 mm, is still debated since it has been questioned by several authors [2]. As for the definition itself, the optimal infertility treatment for women with DIE is not universally agreed upon [3,4].

Current European Society of Human Reproduction and Embryology (ESHRE) guidelines recommend that operative laparoscopy for DIE may be a therapy option for symptomatic individuals who want to get pregnant despite the lack of convincing evidence and despite the potential risk of complications and post-operative adverse effects [5,6,7,8].

Patients with no symptoms do not need to be treated. Hormonal treatments could help with the pain and digestive issues brought on by bowel endometriosis, whereas no infertility is reported [9]. Surgery is commonly indicated for women with occlusive symptoms, those whose symptoms do not improve despite hormonal treatments, those who have contraindications to use hormonal therapies, and those who wish to become pregnant [6].

The involvement of the bowel is often retrievable in women diagnosed with DIE. Various surgical procedures can be used to treat bowel endometriotic nodules. When the endometriotic nodule is removed without first exposing the intestinal lumen, the procedure is referred to as “shaving”; the gut wall may then need to be sutured with staples or sutures. Rectal lesions are often treated by shaving [10,11]. The term “discoid excision” refers to a full-thickness resection of the intestinal wall, which opens the bowel lumen and invariably necessitates suturing of the bowel wall. Segmental resection is the removal of a portion of the bowel followed by an anastomosis [6].

Several studies have demonstrated that bowel involvement impairs fertility and that endometriosis excision in its entirety improves fertility chances [12,13,14,15]. Ballester et al. [15] reported that women with colorectal endometriosis were more subjected to achieving pregnancy after assisted reproduction techniques (ARTs), with higher pregnancy rates when ARTs were employed [15]. Similarly, Stepniewska et al. [14] showed that women with endometriosis-related infertility appeared to have poorer reproductive outcomes when endometriosis invaded their bowel. In terms of post-operative fertility, full endometriosis excision combined with bowel segmental resection appeared to provide superior outcomes [15].

Endometriosis has been linked to an increased risk of pregnancy (including preterm delivery, placenta previa, and possible pre-eclampsia) and non-pregnancy-related complications (including spontaneous hemoperitoneum and bowel perforation), according to earlier research [16]. There is a dearth of information on delivery and neonatal outcomes in women with bowel endometriosis, in contrast to data on infertility and diseases connected to pregnancy.

This retrospective analysis was conducted to assess pregnancy characteristics and complications, labor, and adverse feto-neonatal outcomes in women treated for bowel endometriosis, comparing such patients to matched controls with no diagnosis of superficial or deep endometriosis.

## 2. Materials and Methods

This study was designed as a retrospective analysis of prospectively collected data retrieved from surgical procedures performed for bowel endometriosis at the University Medical Centre of Ljubljana, Department of Human Reproduction, from 1 January 2015, to 31 December 2021.

The study procedure complied with the Committee on Publication Ethics (COPE) standards. The protocol’s design, data collection, analysis, and interpretation procedures, as well as its first draft and any future updates, all adhered to the Strengthening the Reporting of Observational Studies in Epidemiology (STROBE) Statement’s reporting requirements for cohort studies [17]. The obtained data were anonymized to remove any information that may be used to formally identify the patient. Each participant in this study received information about the methods and provided their written consent to allow data to be collected and analyzed for research. The Republic of Slovenia’s Medical Ethics Committee approved the study with reference no. 0120-174/2018/6. We carried out our research according to the Helsinki Declaration of 1975 and its later amendments.

All the women signed a written informed consent before undergoing any study-specific procedure.

Privacy of the participants of this research was maintained for all the study periods. Written informed consent to publication was signed by every woman of this study.

### 2.1. Inclusion and Exclusion Criteria

We included reproductive-aged women (aged 22 to 41) with pregnancy desire who had undergone laparoscopic-assisted surgery for bowel endometriosis.

Every included woman displayed at least one symptom related to bowel endometriosis, including dyschezia, constipation, diarrhea, discomfort during feces, and cyclic rectal bleeding. Three expert operators (Eda Vrtacnik Bokal, Helena Ban Frangez, and Vesna Salamun) made a clinical and transvaginal ultrasound diagnosis of DIE with bowel involvement. Clinical diagnosis was based on the following criteria: infiltration associated with palpable induration on vaginal and rectal digital examinations involving the rectum, rectosigmoid junction associated with vaginal, uterosacral ligaments, or torus uterinum infiltration. Ultrasound diagnosis was based on the diagnosis of hypoechoic nodules in the rectovaginal septum or rectosigmoid colon. In doubtful cases, the suspicion of the disease was confirmed by magnetic resonance imaging (MRI) [18,19,20].

Removal of bowel endometriosis was performed using a laparoscopic approach. According to the size of the lesion, the infiltration of the gut muscularis, the presence of multiple nodules, rectal shaving, discoid resection, or segmental resection were used for treating the pathology [21,22]. All the procedures were carried out with the intention of removing all the visible parts of the nodule. In addition, the procedures also included surgery for additional DIE nodules, ovarian cystectomy or salpingo-oophorectomy, ovarian endometrioma stripping, and uterosacral ligament resection if needed [23]. Women aged less than 22 and more than 41, those with no childbearing desire, women who were deemed unsuitable for or declined surgery, women who had a preoperative diagnosis of current malignant disease, either gynecological or non-gynecological or had severe systemic illnesses (such as autoimmune or endocrine diseases, severe coagulopathy, or cardiac pathology), women who had an acute or chronic genital or urinary tract infection were excluded from the study.

Therefore, endometriosis patients were matched in a 1:3 ratio with healthy controls. Controls were defined included as follows: women had to be statistically similar and matched to endometriotic patients for age, body mass index (BMI), parity (either nulliparous or >1), and gestational age. To be defined as healthy, women in the control group had to have no history or recent anamnesis of gynecological problems, signs and symptoms before pregnancy, no ultrasound signs of superficial, ovarian or deep endometriosis, and no previous laparoscopic or laparotomic gynecological or obstetric (e.g., previous cesarean section) surgery. Moreover, patients had to be included in the Slovenian Perinatal Registry with no missing data.

### 2.2. Study Variables

The following data from endometriosis cases and healthy controls were retrospectively recorded from the patient’s charts: epidemiological characteristics, pregnancy, and maternal and neonatal labor outcomes were collected. Slovenian guidelines were followed in the management of labor and delivery.

Co-primary outcomes for this study were the incidence of labor abnormalities (either dilation or arrest) and cesarean section (CS) rate. Co-secondary outcomes were the incidence of pregnancy complications, labor complications, and the incidence of feto-neonatal complications.

### 2.3. Sample Size

Regarding the number of patients needed to assess the primary outcome, according to the available literature [16,24], assuming two unbalanced groups, a sample size of 201 patients achieved 95% of the power with a significance level of 5% to detect a minimum difference of 10% of the anticipated labor abnormalities incidence between the two groups.

### 2.4. Statistical Analysis

Statistical analysis was performed using Statistical Package for Social Sciences (SPSS) v. 20.0 (IBM Inc., Armonk, NY, USA). Data were shown as mean with standard deviation (SD) for continuous variables or number (percentage) for categorical variables. In addition, a Chi-square or Fisher’s test was conducted for categorical variables and a *t*-test for the comparison of the means of the two groups. All the analyses were performed using a two-sided model, considering a normal distribution as appropriate. We matched patients and used a Cox proportional hazards model to determine the parity, age, BMI and gestational age-adjusted relative risk (aRR), with a 95% confidence interval (CI) for pregnancy and neonatal outcomes. A *p*-value (*p*) of less than 0.05 was considered statistically significant.

## 3. Results

Seventy-one pregnancies from women who had bowel endometriosis surgery were documented over the research period and matched to 213 controls (Table 1).

The women’s mean ages, BMIs, gestational age, and parity were similar to controls (Table 1). Parity, as well as number of pregnancies, due to study design, did not affect the evaluation of the outcomes between the two cohorts (aRR 1.00 [95% CI 0.49–2.01]; *p* = 0.979 and aRR 1.00 [95% CI 0.41 to 2.43]; *p* = 0.998 respectively).

Of those, the number of patients requiring IVF/ET for achieving pregnancy was significantly higher in women with bowel endometriosis (43.7% vs. 11.7%; *p* < 0.001) (Table 1).

Among women with bowel endometriosis, eight out of 71 (11.3%) underwent shaving of the nodule, 31 out of 71 (43.7) underwent bowel resection, and one out of 71 (1.4%) appendectomy for appendicular endometriosis; bowel mobilization at the level of rectovaginal septum was performed in 20 cases (28.2%), while in 11 cases (15.5%), only adhesiolysis (no resection) was performed.

### 3.1. Pregnancy Outcomes

Table 2 shows the pregnancy outcomes between endometriosis and the control group. There was a slightly increased incidence of labor abnormalities with an overall increased risk compared with controls (21.1% vs. 17.4%; *p* = 0.048; aRR 1.36 [95% CI 1.00–1.86]).

The incidence and risk of non-cephalic fetal presentation (14.1% vs. 6.1%; *p* = 0.016; aRR 3.08 [95% CI 2.03–4.68]), CS (43.7% vs. 24.9%; *p* = 0.003; aRR 1.75 [95% CI 1.23–2.49]), and emergent CS (19.7% vs. 8.5%; *p* = 0.009; aRR 2.21 [95% CI 1.55–3.16]) were higher in patients treated for bowel endometriosis compared with controls.

Table 3 depicts a subgroup analysis according to the type of bowel endometriosis surgery.

### 3.2. Pregnancy Complications

Regarding pregnancy complications, the incidence and risk of placenta previa (9.9% vs. 0.0%; *p* < 0.001; aRR 21.82 [95% CI 2.19–116.40]), second-trimester hemorrhage (5.6% vs. 0.9%; *p* = 0.017; aRR 6.00 [95% CI 1.12–32.06]), postpartum hemorrhage (15.5% vs. 3.3%; *p* < 0.001; aRR 4.71 [95% CI 1.90–11.70]), and the need for transfusion during labor (5.6% vs. 0.5%; *p* = 0.004; aRR 12.00 [95% CI 1.36–105.60]) were significantly increased in women with bowel endometriosis compared with healthy women (Table 4).

There were no significant differences according to the surgical approach involved (Table 5).

### 3.3. Feto-Neonatal Complications

Table 4 shows the comparison between bowel endometriosis patients and healthy matched controls for neonatal outcomes. An increased risk for neonatal intensive care unit (NICU) admission in favor of newborns of endometriotic women (26.0% vs. 6.9%; *p* < 0.001; aRR 3.75 [2.04–3.86]) was calculated. No other significant differences were noted (Table 6).

No differences regarding feto-neonatal complications according to the type of bowel surgery used in the 31 cases were notable (Table 7).

## 4. Discussion

This retrospective cohort study showed that women treated for bowel endometriosis were exposed to several increased risks for adverse obstetric and perinatal outcomes, including placenta previa, labor abnormalities, non-cephalic fetal presentations, increased risk of planned or emergent CS, second-trimester and postpartum hemorrhage, and increased need for transfusion, as well as higher neonatal intensive care unit (NICU) access compared with parity, age, BMI and gestational age-matched healthy controls.

A large registry analysis from Farland et al. [25] reported that preterm birth, pregnancy-induced hypertension, pre-eclampsia, eclampsia, cesarean delivery, and placental abnormalities were more common in women with endometriosis (regardless of infiltration or location) or fibroids. Additionally, there was a higher likelihood of low birthweight and respiratory issues among children delivered by mothers who had previously been diagnosed with endometriosis or fibroids [25]. Such data were confirmed in a recent meta-analysis reporting that, regardless of location, an increased risk of gestational hypertension, pre-eclampsia, preterm birth, placenta previa, placental abruption, cesarean section, and stillbirth was retrievable in women with endometriosis compared with women without endometriosis [26].

It has been established that endometriosis-affected women’s fertility may be significantly impacted by the existence of intestinal lesions. Endometriosis-related pelvic inflammation also contributes to reproductive impairment [16,24,25].

Remarkably, our study is the first to compare women with bowel endometriosis with paired controls with the same parity, age, BMI, and gestational age in order to have valid comparability of the two cohorts. In our study of patients with bowel endometriosis, 57 out of 71 (80.2%) were diagnosed with infertility and became pregnant after surgery. Even if there was a higher incidence of IVF pregnancies compared with control, underlining the impaired fertility of women with such a disease, it should also be highlighted that more than half of the women became pregnant spontaneously, strengthening the evidence that bowel endometriosis alone is a risk factor for infertility and that surgery could help to resolve the issue.

Previous studies highlighted the higher pregnancy rates when ARTs were utilized in women with colorectal endometriosis [15,27]. Moreover, when bowel invasion was noted, full endometriosis excision combined with bowel segmental resection was able to increase reproductive outcomes [12,15].

Stepniewska et al. [14] stressed the significance of preoperative bowel assessment in infertile patients with severe endometriosis. When weighing the potential dangers and advantages of surgery in these situations, the improvement in post-operative fecundity should be considered in the decision-making process for therapeutic treatment, which remains associated with risks and complications [12,14,24].

However, data regarding obstetric outcomes for bowel endometriosis patients alone are still lacking in current literature. To date, only limited retrospective analyses with no control groups could be retrieved. Patients with bowel endometriosis were exposed to adverse obstetric outcomes even in a retrospective study by Thomin et al. [24]. Even with the strong limitation of the absence of a control group, they showed that, despite previous resection surgery, a substantial risk of increased cesarean delivery was available. In addition, especially in women with anterior DIE, a significant incidence of difficult extraction during CS was seen. As also reported in our results, in women with colorectal endometriosis, vaginal birth had a high rate of surgical interventions, and both cesarean section and vaginal birth had higher rates of postpartum diseases [24].

There are currently no guidelines for the management of labor after previous bowel surgery. In our study, the higher number of CS and, foremost, the increased risk of emergency CS was related to labor abnormalities, non-cephalic fetal presentation, and mainly to the presence of placenta previa. The latter may indicate the presence of undiagnosed adenomyosis occurring as part of DIE. These findings are shared with Exacoustos et al. [28]. They found that the number of CS was higher in women with posterior DIE compared with healthy controls, with increased complications during surgery, including hysterectomy, hematoperitoneum and bladder damage. Additionally, they described a ten-fold higher risk of placenta previa in women with DIE [28]. Nirgianakis et al. [29] added that even the surgical removal of endometriotic nodules did not reduce the risk of placenta previa and pregnancy complications [29].

Moreover, the higher incidence of placenta previa and other adverse obstetric outcomes could also be related to the presence of adenomyosis, which occurs more often together with other forms of endometriosis, especially DIE, and may be unrecognized or less diagnosed [30].

The association of adenomyosis with poor obstetric outcomes has already been extensively reported [30,31,32]. In fact, uterine dysperistalsis, poor or abnormal myometrial contractility due to the presence of adenomyotic tissue, can lead to abnormalities during labor and, consequently, to increased emergency CS, malpresentation, and postpartum hemorrhage [33,34].

The presence of labor abnormalities, alongside other obstetrical complications, could also be the compelling reason for the increased risk of NICU admission in newborns of patients with bowel endometriosis. Conti et al. [35] highlighted the evidence that newborns of primiparous patients with endometriosis were exposed to more NICU admissions and longer hospitalization, particularly related to high incidence of FGR and preterm delivery [35]. It should be underlined that the significant increase in the neonatal hospitalization rate, in our experience, was not associated with the increase in prematurity since similar gestational age, number of FGR/SGA babies and low APGAR scores were reported. Therefore, such evidence deserves further investigation to clarify the rationale behind endometriosis and raised NICU admissions.

Several limitations should be accounted for in this study. Firstly, there could be inherent biases related to its retrospective design. However, the use of prospectively collected data might restrain such issues. Secondly, although reaching the minimum sample size and being one of the extremely limited numbers of studies on the research topic, the amount of bowel endometriosis women should be considered low with only a single center referring data, limiting the generalization of the findings, and stressing the need for additional comparative studies. Thirdly, measures of risks had wide CIs for a reasonably low number of selected outcomes (placenta previa, preterm birth, APGAR < 5 at 5 min, severe neonatal asphyxia). This was related to the extremely low incidence of such conditions both in the study and control group, increasing the need for wider studies to draw firmer conclusions. Moreover, due to the reduced number of cases among the bowel endometriosis cohort, the findings of the subgroup analysis according to the surgical technique are consequentially limited in robustness and generalizability. Lastly, we were not able to assess the co-presence of adenomyosis and bowel endometriosis, which inherently would have strengthened the reasons for poor obstetric outcomes in the endometriosis cohort.

Nonetheless, this study has several strengths. Firstly, it is the first to summarize in terms of risk estimation the pregnancy and neonatal outcomes of women with bowel endometriosis, while most of the available literature takes in consideration every location of the disease or generally the involvement of anterior or posterior DIE. Secondly, the accuracy of data reporting in our electronic system, with no missing data at the analysis, increases the robustness of findings.

## 5. Conclusions

Despite the limitations derived from the study design, we reported that, compared with healthy women without endometriosis, women subjected to surgery for bowel endometriosis, together with having an increased need for ART, showed an increased risk of worsened pregnancy and neonatal outcomes, especially placenta previa and labor abnormalities, non-cephalic fetal presentations, increased risk of emergent CS, and second-trimester and postpartum hemorrhage, need for transfusion after delivery and increased NICU access for the newborn.

Additional studies with a prospective design are needed to further confirm the available evidence showing that endometriosis, even if in non-gynecological localizations, raises the risks associated with pregnancy and labor to draw guidelines on the topic.

## Figures and Tables

**Table 1:** Baseline characteristics of women treated for bowel endometriosis and matched controls.

	Endometriosis	Controls	*p*-Value
Age, years (SD)	32.06 (3.31)	32.08 (3.29)	0.967
BMI, Kg/m^2^ (SD)	22.48 (3.72)	22.51 (3.76)	0.960
Gestational age, weeks (SD)	37.01 (3.69)	38.77 (2.22)	0.891
Parity, *n* (%)			0.999
0	62 (87.3)	188 (87.3)	
>1	9 (12.7)	27 (12.7)	
Conception, *n* (%)			<0.001
Spontaneous	40 (56.3)	186 (87.3)	
IVF/ET	31 (43.7)	25 (11.7)	
Ovulation induction	0 (0)	2 (0.9)	
Pregnancies, *n* (%)			0.370
Singleton	65 (91.5)	195 (91.5)	
Twin:			
Bichorial-biamniotic	4 (5.6)	14 (6.6)	
Monochorial-biamniotic	1 (1.4)	4 (1.9)	
Monochorial-monoamniotic	1 (1.4)	0 (0)	

SD: standard deviation; BMI: body mass index; IVF/ET in vitro fertilization/embryo transfer.

**Table 2:** Labor outcomes between bowel endometriosis cases and matched controls.

	Endometriosis	Controls	*p*-Value	aRR
Fetal presentation, *n* (%)			0.016	3.08 (2.03–4.68)
Cephalic	61 (85.9)	200 (93.9)		
Non-cephalic:				
Transverse	8 (11.3)	13 (6.1)		
Breech	2 (2.8)	0 (0)		
Labor abnormalities, *n* (%)			0.040	1.39 (1.06–2.05)
Dystocia	2 (9.8)	16 (7.5)		
Arrest	2 (11.3)	21 (9.9)		
Mode of delivery, *n* (%)			0.003	1.75 (1.23–2.49)
Vaginal	40 (56.3)	160 (75.1)		
Cesarean Section	31 (43.7)	53 (24.9)		
Epidural anesthesia, *n* (%)	17 (23.9)	45 (21.1)	0.619	1.13 (0.69–1.84)

**Table 3:** Labor outcomes according to the type of bowel endometriosis surgical technique.

	Shaving	Resection	Appendectomy	Mobilization	No Resection	*p*-Value
Fetal presentation, *n* (%)						0.820
Cephalic	6 (75)	26 (83.9)	1 (100)	18 (90)	10 (90.1)	
Non-cephalic:						
Transverse	2 (25)	3 (9.7)	0 (0)	2 (10)	1 (9.9)	
Breech	0 (0)	2 (6.5)	0 (0)	0 (0)	0 (0)	
Labor abnormalities, *n* (%)						0.301
Dystocia	0 (0)	1 (3.2)	0 (0)	0 (0)	1 (9.1)	
Arrest	0 (0)	1 (3.2)	0 (0)	0 (0)	1 (9.1)	
Mode of delivery, *n* (%)						0.212
Vaginal	3 (37.5)	17 (54.8)	0 (0)	15 (75)	5 (45.5)	
Cesarean Section	5 (62.5)	14 (45.2)	1 (100)	5 (25)	6 (54.5)	
Epidural anesthesia, *n* (%)	2 (11.8)	7 (41.2)	0 (0)	5 (29.4)	3 (17.6)	0.096

**Table 4:** Pregnancy complications between bowel endometriosis cases and matched controls.

	Endometriosis	Controls	*p*-Value	aRR
Emergency CS, *n* (%)	14 (19.7)	18 (8.5)	0.009	2.21 (1.55–3.16)
Vacuum use, *n* (%)	1 (1.4)	9 (3.9)	0.302	0.36 (0.04–1.77)
Episiotomy, *n* (%)	20 (28.2)	73 (34.3)	0.343	0.82 (0.54–1.24)
Pre-eclampsia, *n* (%)	3 (4.2)	3 (2.1)	0.153	3.00 (0.61–14.53)
Gestational Hypertension, *n* (%)	6 (8.5)	7 (3.3)	0.071	2.57 (0.89–7.40)
Early pregnancy loss (<12 weeks), *n* (%)	10 (14.1)	39 (18.3)	0.414	0.77 (0.40–1.45)
Preterm birth (<34 weeks), *n* (%)	1 (1.4)	1 (0.5)	0.563	2.99 (0.36–21.17)
Postpartum hemorrhage (>500 mL), *n* (%)	11 (15.5)	7 (3.3)	<0.001	4.71 (1.90–11.70)
Abruptio placentae, *n* (%)	1 (1.4)	3 (0.9)	0.738	1.00 (0.11–9.50)
Placenta previa, *n* (%)	7 (9.9)	0 (0)	<0.001	21.82 (2.19–116.40)
1st-trimester hemorrhage, *n* (%)	6 (8.5)	13 (6.7)	0.494	1.38 (0.54–3.50)
2nd-trimester hemorrhage, *n* (%)	4 (5.6)	2 (0.9)	0.017	6.00 (1.12–32.06)
3rd-trimester hemorrhage, *n* (%)	2 (2.8)	4 (1.9)	0.634	1.50 (0.38–8.02)
Need for transfusion, *n* (%)	4 (5.6)	1 (0.5)	0.004	12.00 (1.36–105.60)
Chorioamnionitis	1 (1.4)	0 (0)	0.143	5.94 (0.55–64.59)

CS: cesarean section.

**Table 5:** Pregnancy complications among bowel endometriosis-related surgical approaches.

	Shaving	Resection	Appendectomy	Mobilization	No Resection	*p*-Value
Emergency CS, *n* (%)	2 (25)	8 (25.8)	0 (0)	1 (5)	3 (27.3)	0.412
Vacuum use, *n* (%)	0 (0)	1 (3.2)	0 (0)	0 (0)	0 (0)	0.961
Episiotomy, *n* (%)	2 (25)	11 (35.5)	0 (0)	3 (15)	4 (36.5)	0.504
Pre-eclampsia, *n* (%)	1 (12.5)	1 (3.2)	0 (0)	0 (0)	1 (9.1)	0.560
Gestational Hypertension, *n* (%)	1 (12.5)	3 (9.7)	0 (0)	1 (5)	1 (9.1)	0.971
Early pregnancy loss (<12 weeks), *n* (%)	2 (25)	7 (22.6)	0 (0)	0 (0)	1 (9.1)	0.525
Preterm birth (<34 weeks), *n* (%)	0 (0)	1 (3.2)	0 (0)	0 (0)	0 (0)	0.961
Postpartum hemorrhage (>500 mL), *n* (%)	0 (0)	8 (25.8)	0 (0)	3 (15)	0 (0)	0.185
Abruptio placentae, *n* (%)	0 (0)	1 (3.2)	0 (0)	0 (0)	0 (0)	0.961
Placenta previa, *n* (%)	0 (0)	6 (19.4)	0 (0)	0 (0)	1 (9.1)	0.176
1st-trimester hemorrhage, *n* (%)	1 (12.5)	3 (9.7)	0 (0)	1 (5)	1 (9.1)	0.953
2nd-trimester hemorrhage, *n* (%)	0 (0)	3 (9.7)	0 (0)	1 (5)	0 (0)	0.706
3rd-trimester hemorrhage, *n* (%)	0 (0)	0 (0)	0 (0)	2 (10)	0 (0)	0.263
Need for transfusion, *n* (%)	0 (0)	3 (9.7)	0 (0)	1 (5)	0 (0)	0.706
Chorioamnionitis, *n* (%)	0 (0)	1 (3.2)	0 (0)	0 (0)	0 (0)	0.961

CS: cesarean section.

**Table 6:** Feto-neonatal complications for cases and matched controls.

	Endometriosis	Controls	*p*-Value	aRR
SGA or FGR, *n* (%)	12 (15.6)	23 (10)	0.178	1.56 (0.82–2.99)
NICU access, *n* (%)	20 (26)	16 (6.9)	<0.001	3.75 (2.04–6.86)
APGAR < 5 1 min, mean (SD)	2 (2.6)	4 (1.7)	0.634	1.50 (0.28–8.03)
APGAR < 5 5 min, mean (SD)	1 (1.3)	0 (0)	0.083	5.89 (0.64–44.12)
Stillbirths, *n* (%)	2 (2.6)	2 (0.9)	0.245	3.00 (0.43–20.94)
Severe asphyxia, *n* (%)	0 (0)	1 (0.5)	0.563	0.99 (0.04–24.59)

NE: not estimable; NICU: neonatal intensive care unit. SGA: small for gestational age; FGR: fetal growth restriction; aRR: adjusted risk ratio; SD: standard deviation.

**Table 7:** Feto-neonatal complications for each type of bowel surgery.

	Shaving	Resection	Appendectomy	Mobilization	No Resection	*p*-Value
SGA or FGR, *n* (%)	2 (25)	6 (19.3)	0 (0)	3 (15)	1 (9.1)	0.778
NICU access, *n* (%)	2 (25)	15 (40.5)	0 (0)	1 (5)	2 (18.2)	0.053
APGAR <5 1min, mean (SD)	0 (0)	1 (3.2)	0 (0)	1 (5)	0 (0)	0.911
APGAR <5 5min, mean (SD)	0 (0)	1 (2.7)	0 (0)	0 (0)	0 (0)	0.895
Stillbirths, *n* (%)	0 (0)	1 (3.2)	0 (0)	0 (0)	1 (9.1)	0.758

NE: not estimable; NICU: neonatal intensive care unit. SGA: small for gestational age; FGR: fetal growth restriction; aRR: adjusted risk ratio; SD: standard deviation.

## Data Availability

The original contributions presented in the study are included in the article; further inquiries can be directed to the corresponding author.
